# Respiratory compliance but not gas exchange correlates with changes in lung aeration after a recruitment maneuver: an experimental study in pigs with saline lavage lung injury

**DOI:** 10.1186/cc3772

**Published:** 2005-07-13

**Authors:** Dietrich Henzler, Paolo Pelosi, Rolf Dembinski, Annette Ullmann, Andreas H Mahnken, Rolf Rossaint, Ralf Kuhlen

**Affiliations:** 1 Senior Anesthesiologist, Anesthesiology Department, University Hospital RWTH Aachen, Germany; 2 Professor of Anesthesiology, Environment, Health and Safety Department, University of Insubria, Varese, Italy; 3 Intensivist, Surgical Intensive Care Department, University Hospital RWTH Aachen, Germany; 4 Resident, Anesthesiology Department, University Hospital RWTH Aachen, Germany; 5 Department of Clinical Radiology, University Hospital RWTH Aachen, Germany; 6 Professor of Anesthesiology, Anesthesiology Department, University Hospital RWTH Aachen, Germany; 7 Head, Surgical Intensive Care Department, University Hospital RWTH Aachen, Germany

## Abstract

**Introduction:**

Atelectasis is a common finding in acute lung injury, leading to increased shunt and hypoxemia. Current treatment strategies aim to recruit alveoli for gas exchange. Improvement in oxygenation is commonly used to detect recruitment, although the assumption that gas exchange parameters adequately represent the mechanical process of alveolar opening has not been proven so far. The aim of this study was to investigate whether commonly used measures of lung mechanics better detect lung tissue collapse and changes in lung aeration after a recruitment maneuver as compared to measures of gas exchange

**Methods:**

In eight anesthetized and mechanically ventilated pigs, acute lung injury was induced by saline lavage and a recruitment maneuver was performed by inflating the lungs three times with a pressure of 45 cmH_2_O for 40 s with a constant positive end-expiratory pressure of 10 cmH_2_O. The association of gas exchange and lung mechanics parameters with the amount and the changes in aerated and nonaerated lung volumes induced by this specific recruitment maneuver was investigated by multi slice CT scan analysis of the whole lung.

**Results:**

Nonaerated lung correlated with shunt fraction (r = 0.68) and respiratory system compliance (r = 0.59). The arterial partial oxygen pressure (PaO_2_) and the respiratory system compliance correlated with poorly aerated lung volume (r = 0.57 and 0.72, respectively). The recruitment maneuver caused a decrease in nonaerated lung volume, an increase in normally and poorly aerated lung, but no change in the distribution of a tidal breath to differently aerated lung volumes. The fractional changes in PaO_2_, arterial partial carbon dioxide pressure (PaCO_2_) and venous admixture after the recruitment maneuver did not correlate with the changes in lung volumes. Alveolar recruitment correlated only with changes in the plateau pressure (r = 0.89), respiratory system compliance (r = 0.82) and parameters obtained from the pressure-volume curve.

**Conclusion:**

A recruitment maneuver by repeatedly hyperinflating the lungs led to an increase of poorly aerated and a decrease of nonaerated lung mainly. Changes in aerated and nonaerated lung volumes were adequately represented by respiratory compliance but not by changes in oxygenation or shunt.

## Introduction

Severe impairment of oxygenation in acute lung injury and in the acute respiratory distress syndrome (ARDS) is caused by an inhomogenous ventilation-perfusion distribution () and an increase in shunt fraction. The amount of aerated lung is markedly reduced due to alveolar collapse and flooding [1,2]. Mechanical ventilation has been shown to further aggravate the  mismatch [3]. Even though it is unclear if the optimal treatment should aim to improve gas exchange, to prevent additional lung damage or to resolve the existing damage, one of the commonly used treatment concepts is the open-lung approach [4], aiming at recruitment and maintenance of ventilated lung volume. In general, recruitment means to transform nonaerated into aerated lung. These regions can open and close or can be kept opened if sufficient positive endexpiratory pressure (PEEP) is applied. Significant controversy exists over the optimal method to achieve alveolar recruitment and to the definition of recruitment, whether it means re-opening of collapsed alveoli or edema clearance [2]. Improvement in oxygenation is commonly used to detect recruitment, although gas exchange is also influenced by many other factors, like ventilation-perfusion distribution, pulmonary blood flow and regional vascular regulation [5,6]. The assumption that the gas exchange parameters adequately represent the mechanical process of alveolar opening has not been proven so far. The best available technique to detect recruitment is computed lung tomography [7] where the decrease of atelectatic lung can be visualized [8]. Since computer tomographic (CT) scanning cannot be performed repeatedly under clinical conditions, different parameters must be obtained at the bedside in order to indicate successful recruitment. The aim of this study was to investigate whether commonly used measures of lung mechanics better detect lung tissue collapse and changes in lung aeration after a recruitment maneuver as compared to measures of gas exchange.

## Materials and methods

After governmental approval, eight anesthetized female pigs (31.3 ± 1.9 kg) were orotracheally intubated and ventilated in constant flow mode with a fraction of inspired oxygen (FiO_2_) of 1.0, a tidal volume of 8 ml/kg with an inspiratory-expiratory (I:E) ratio of 1:1 and PEEP of 10 cmH_2_O throughout the study. Deep anesthesia was maintained with a continuous infusion of propofol (7.7 ± 1.7 mgkg^-1^h^-1^) and fentanyl (8.0 ± 2.2 μgkg^-1^h^-1^) and animals were additionally paralyzed with pancuronium (0.3 ± 0.1 mgkg^-1^h^-1^) for the actual experimental phase. Handling of animals conferred to the guidelines laid out in the Guide for the Care and Use of Laboratory Animals [9].

Arterial and pulmonary artery catheters (Becten Dickinson, Heidelberg, Germany) were placed and cardiac output was determined through thermodilution with equipment from Datex-Ohmeda (Duisburg, Germany). The extravascular lung water index was determined by transcardiopulmonary thermodilution with equipment from Pulsion (Munich, Germany). Gas flow and airway pressures were measured at the proximal end of the tracheal tube. The esophageal pressure was measured using a balloon catheter (International Medical, c/o Allegiance, Kleve, Germany). Expiratory volumes were corrected as described previously [10]. A more detailed description can be found in Additional file 1.

### Experimental protocol

Acute lung injury was induced through repeated lung lavage as described previously [11] and allowed to stabilize until the arterial blood partial oxygen pressure (PaO_2_) had been below 100 mmHg for 60 minutes. The following measurements were obtained before and 10 minutes after a recruitment maneuver was performed.

#### Lung volumes

Contiguous multi-slice CT scans of the whole lung (Siemens Sensation 16, Forchheim, Germany) were taken at end-expiratory and end-inspiratory occlusion [1,12]. From the reconstructed slices (2 mm) the lung was delineated by hand from the inner pleura. The calculations for hyperinflated parenchyma (HYP; -1000 to -900 Hounsfield units (HU)), normally aerated (NORM; -900 to -500 HU), poorly aerated (POOR; -500 to -100 HU) and non-aereated parenchyma (NON; -100 to +100 HU) were done by the CT software with a pixel size of 0.59 mm. The resulting areas were multiplied with the slice thickness and then added together for lung volumes (V_TOT_, V_HYP_, V_NORM_, V_POOR_, V_NON_). The intrathoracic gas volume was calculated as V_GAS _= V_TOT _× HU_MEAN_/-1000 and the intrathoracic tissue volume was calculated as V_TISS _= V_TOT _- V_GAS_. The lung volumes consisted of V_GAS _+ V_TISS_, for example, a mean HU of -500 representing 50% gas and 50% tissue. Recruitment was defined as a decrease in the nonaerated lung volume after the recruitment maneuver [13].

#### Venous admixture and dead space

Arterial and mixed venous blood samples were collected simultaneously and analyzed immediately using equipment by Radiometer, Copenhagen, Denmark. Venous admixture (Q_VA_/Q_T_) was calculated using the shunt equation [14] and dead space (V_D_/V_T_) according to the modified Bohr equation.

#### Compliance of the respiratory system

The static compliance of the respiratory system (C_RS_) was computed using the occlusion technique [15].

#### Inflation compliance and recruitable volume

An inflation-deflation pulmonary pressure-volume curve (PV-curve) starting from zero end-expiratory pressure (ZEEP) was performed using a new tool that was built into the ventilator (Galileo Gold, Hamilton, Rhäzüns, Switzerland). Objective analysis of inflation and deflation curves was performed by fitting it to the Venegas-Harris equation [16]. The corner points stating the point of maximum compliance increase and decrease, being the mathematical equivalents of lower and upper inflection points, were calculated. The maximum inflation compliance (C_INF_) was calculated through numerical differentiation of the true inflection point. The recruitable volume (V_REC_) was defined as the end-expiratory volume difference between the inflation and deflation pressure obtained at PEEP level (10 cmH_2_O).

The actual recruitment maneuver was performed by inflating the lungs three times with a pressure of 45 cmH_2_O for 40 s [8,17-19], with 10 normal tidal breaths between inflations. A detailed description of animal preparation and measurements can be found in Additional file 1. After the experiment, the animals were killed with a barbiturate overdose.

### Statistical analysis

All data are reported as mean ± SD. To correlate the parameters under investigation with the CT measurements, the Pearson's coefficient (r) was calculated. Where appropriate, multiple linear regression was used. The validity of the model was verified by a Durbin-Watson statistic. Because correlations of parameters with end-inspiratory or end-expiratory CT measurements exhibited equal results, only the end-expiratory data are presented. To determine the parameter with the strongest influence, the dimensionless standardized beta coefficient (beta_S_) was calculated. Pre- and post-recruitment maneuver (RM) values were compared using Wilcoxon's signed ranks test. In the case of parameters exhibiting a significant difference, the dimensionless fractional change for any parameter 'X' was then calculated as *fractional change* (X) = X_postRM_/X_preRM _- 1 and correlation analysis performed as explained above. Fractional change values are expressed as percentages. Statistical significance was accepted at p < 0.05 (SPSS 11.0, SPSS, Chicago, USA).

## Results

### Correlation of the CT data with gas exchange and respiratory mechanics parameters before and after a recruitment maneuver

#### Parameters correlating with aerated lung

**Figure 1:**
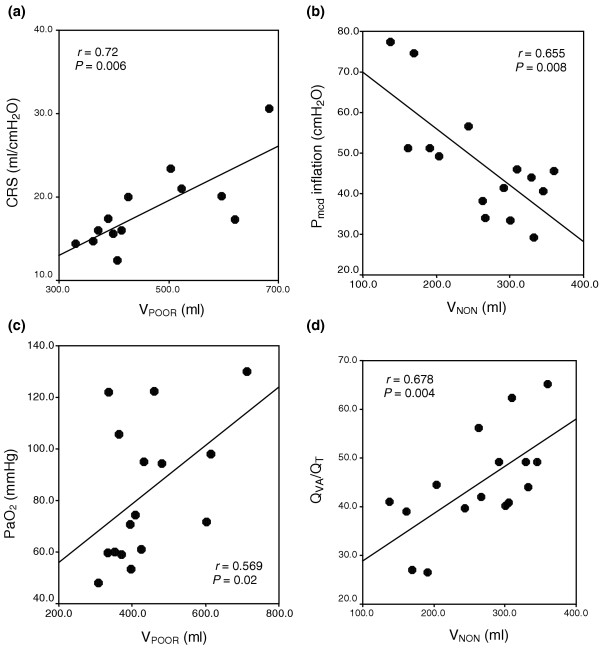
Correlation of expiratory multi-slice CT lung volumes with respiratory mechanics and gas exchange parameters. CRS, static compliance of respiratory system; PaO_2_, arterial partial oxygen pressure; P_mcd_, pressure of maximum compliance decrease on inflation curve; Q_VA_/Q_T_, venous admixture; V_NON_, nonaerated lung volume; V_POOR_, poorly aerated lung volume.

No significant correlations were found between the gas exchange or respiratory mechanics parameters and normally aerated lung volume. Instead, a significant correlation was observed between poorly aerated lung volume and the PaO_2 _(r = 0.569, p = 0.022) (Fig. 1c) and also between V_POOR _and respiratory system compliance (r = 0.719, p = 0.006) (Fig. 1a) and the inflation pressure maximum compliance increase (r = 0.655, p = 0.008).

#### Parameters correlating with nonaerated lung

Venous admixture correlated directly with nonaerated lung volume (r = 0.678, p = 0.004) (Fig. 1d), but the PaO_2 _did not (p = 0.098). Similarly, nonaerated lung volume correlated with physiologic dead space (r = 0.534, p = 0.04), but not with the arterial blood partial carbon dioxide pressure (PaCO_2_; p = 0.154). Of the respiratory mechanics parameters, the respiratory system compliance (r = -0.587, p = 0.035) and the inflation point of maximum compliance decrease (r = -0.77, p = 0.001) correlated with the nonaerated lung volume (Fig. 1b). Multiple regression analysis revealed that the best prediction of nonaerated volume was achieved by a combination of inflation point of maximum compliance decrease (beta_S _= -0.563) and venous admixture (beta_S _= 0.45).

### Effects of the recruitment maneuver

#### CT lung volume measurements

**Figure 2:**
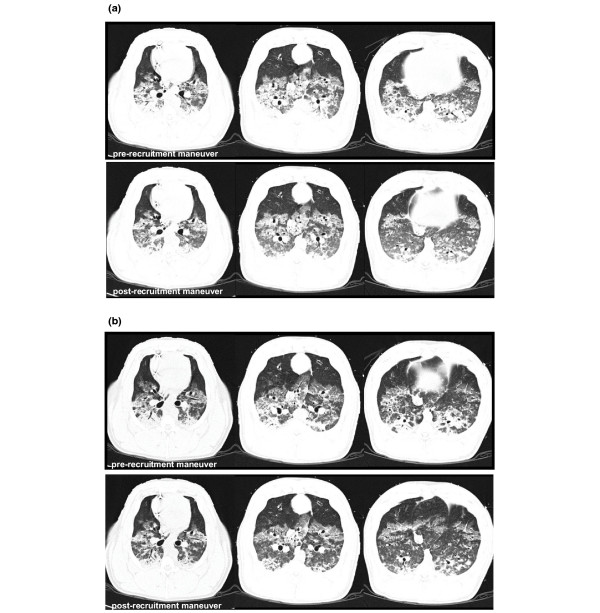
Representative CT scan of one animal at three different levels (apical, middle, basal). **(a) **Expiratory occlusion (10 cmH2O) before and after the recruitment maneuver. Lung volumes in this animal changed as follows: V_HYP _+1%, V_NORM _+15%, V_POOR _+17%, V_NON _-30%, V_GAS _+11%. **(b) **Inspiratory occlusion at plateau pressure before and after the recruitment maneuver. Lung volumes in this animal changed as follows: V_HYP _+6%, V_NORM _+17%, V_POOR _+26%, V_NON _-29%, V_GAS _+17%. V_GAS_, intrathoracic gas volume; V_HYP_, volume of hyperinflated lung parenchyma; V_NON_, volume of nonaerated lung parenchyma; V_NORM_, volume of normally aerated lung parenchyma; V_POOR_, volume of poorly aerated lung parenchyma.

**Figure 3:**
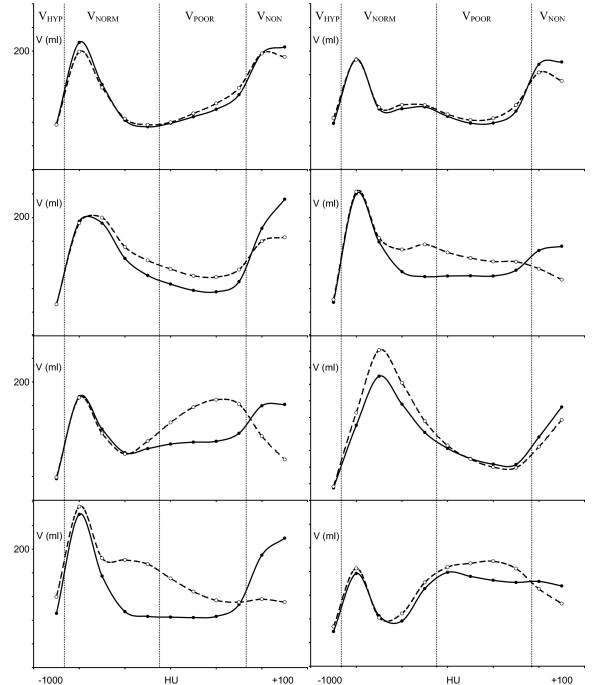
Distribution of differently aerated lung volumes. Individual curves for eight animals before (solid line) and after (dashed line) a recruitment maneuver. Multi-slice CT of the whole lung with characterization of lung parenchyma according to Hounsfield units at end-expiration. V_HYP_, volume of hyperinflated lung parenchyma; V_NON_, volume of nonaerated lung parenchyma; V_NORM_, volume of normally aerated lung parenchyma; V_POOR_, volume of poorly aerated lung parenchyma.

Atelectasis and consolidation were found predominately in the dependent two-thirds of the lung (Fig. 2). The recruitment maneuver caused a significant decrease in nonaerated lung volume by approximately 22% (Table 1). It is important to note that the recruitment was associated with an increase in poorly aerated and normally aerated lung volume. The individual changes in CT lung volumes are shown in Fig. 3. The increase of V_POOR _(21.7%, beta_S _= 0.668) contributed more to recruitment than the increase of V_NORM _(11%, beta_S _= 0.641).

The 13% increase in V_GAS _represents an increase in the functional residual capacity, because the inspiratory-expiratory volume difference did not change (211 ± 33 ml pre-RM versus 221 ± 45 ml post-RM, p = 0.46). No differences in tidal volumes were found between the measurement with CT and spirometry. Importantly, the inspiratory-expiratory volume change in nonaereated regions (62 ± 18 ml), representing opening and collapse of alveoli, was not significantly reduced after the recruitment maneuver (43 ± 26 ml, p = 0.114). The *fractional change *(V_GAS_), however, was not correlated with any parameter of gas exchange or respiratory mechanics; it only correlated with *fractional change *(V_NORM_), which could be expected from recruitment.

#### Effects on gas exchange

**Figure 4:**
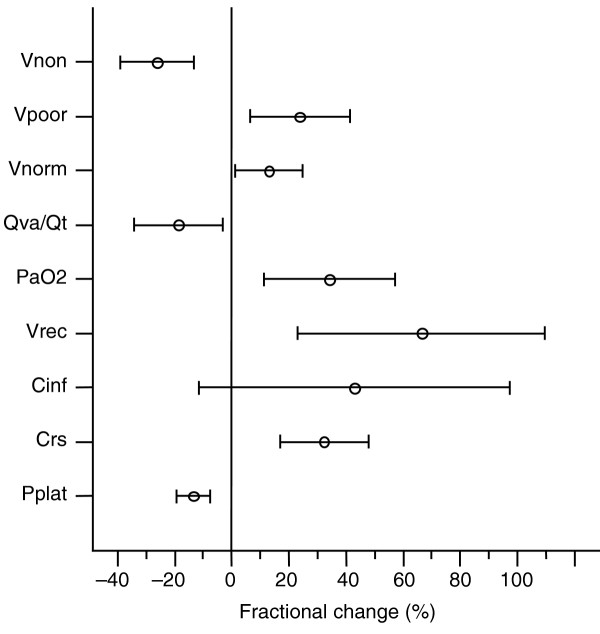
Fractional changes in investigated parameters (means with confidence intervals). Cinf, maximum inflation compliance; Crs, static compliance of respiratory system; PaO_2_, arterial partial oxygen pressure; Pplat, plateau pressure; Q_VA_/Q_T_, venous admixture; V_NON_, nonaerated lung volume; V_NORM_, normally aerated lung volume; V_POOR_, poorly aerated lung volume; Vrec, recruitable volume at PEEP.

**Figure 5:**
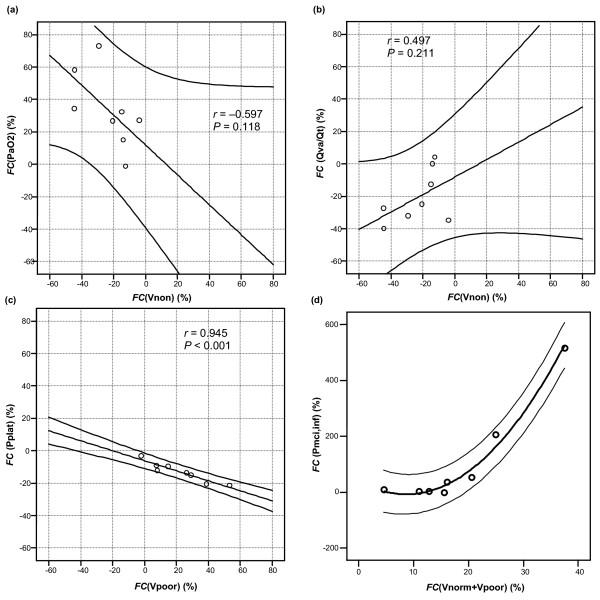
Correlation of the fractional changes (*FC*; %) of parameters with multi-slice CT lung volumes. Regression lines with 95% individual confidence intervals.**(a) **Insignificant correlation of arterial partial oxygen pressure (PaO_2_) with nonaerated lung. Note the large confidence intervals. **(b) **Insignificant correlation of venous admixture (Q_VA_/Q_T_) with nonaerated lung. **(c) **Close relation between changes in plateau pressure (P_PLAT_) and poorly aerated lung. **(d) **Pressure of maximum compliance increase on inflation curve (Pmci) correlates non-linearly with aerated volume (volume of normally aerated lung parenchyma (V_NORM_) + volume of poorly aerated lung parenchyma (V_POOR_)). Note the sharp increase of Pmci beyond 20% increase in aerated lung volume.

The distributions of the fractional changes of the parameters under investigation can be seen in Fig. 4. Overall, a significant improvement in oxygenation (*fractional change *(PaO_2_), +33%) and a shunt reduction (*fractional change *(Q_VA_/Q_T_), -20.8%) were observed (Table 2). The *fractional change *(PaO_2_) did not correlate well with the increase of normally or poorly aerated lung (r = 0.51, p = 0.18), however, nor did the *fractional change *(Q_VA_/Q_T_) correlate with the decrease of nonaerated lung (r = 0.50, p = 0.21) (Fig. 5a,b). No significant changes in PaCO_2 _nor dead space were observed. From these data it seems that the changes in gas exchange parameters do not correlate with the changes in aerated or nonaerated volumes caused by a recruitment maneuver.

#### Effects on respiratory mechanics

In accordance with the CT-measurements, there were no changes in tidal volume, but peak and plateau pressures did decrease (Table 3), which correlated with the *fractional change *(V_NON_) (Fig. 5c). There was a significant increase in compliance and recruitable volume. The increase in C_RS _correlated positively with the increase in poorly aerated lung (r = 0,822, p = 0.012) and inversely with the decrease in nonaerated lung volumes (r = -0.721, p = 0.043). The decrease of nonaerated lung volume could be predicted from the equation *fractional change *(V_NON_) = -0.69 × *fractional change *(C_RS_). This means the decrease of atelectasis can be estimated to be roughly two-thirds of the increase in C_RS_. Interestingly, we found no significant correlations with normally aerated lung volume.

After the recruitment maneuver, the PV-curve was expanded vertically (see Additional file 1; Fig. 4). The resultant increase in the inflational point of maximum compliance increase correlated with the increase in the sum of V_NORM _and V_POOR _(r = 0.914) (Fig. 5d). The fractional changes of V_REC _correlated positively with an increase in V_POOR _(r = 0.863, p = 0.034) and also inversely with a decrease in V_NON _(r = -0.775 (p = 0.041).

#### Effects on hemodynamics

With no changes in sedation and fluid management, only heart rate and cardiac output decreased after the recruitment maneuver. However, no changes in systemic or pulmonary pressures nor vascular resistance could be observed. The extravascular lung water index indicated massive pulmonary edema, but did not change after the recruitment maneuver either (see Additional file 1; Table 2).

In summary, changes in compliance of the respiratory system but not in gas exchange parameters correlated with changes in nonaerated and aerated lung before and after a recruitment maneuver at the same PEEP level of 10 cmH_2_O.

## Discussion

### Experimental considerations

We investigated parameters used to indicate the amount and the change of aerated and nonaerated lung in acute lung injury. We chose the lavage model in pigs for this because it is known to be easily recruitable. This model has been shown to cause lung inflammation [20], ventilation-perfusion mismatch equal to other models [21] and an increase in extravascular lung water and excess tissue [22]. Furthermore, the preferential distribution of atelectasis to the dependent lung could also be demonstrated in patients with ARDS by use of CT scanning [12]. The number of experiments is in line with recent studies investigating respiratory mechanics in acute lung injury [23,24]. Increasing the power may have resulted in more subtle correlations, although we have found some correlations to be significant (certain effect) and others not (possible effect).

Our definition of recruitment may be questioned, because what we measured really is a density scale proportional to gas-tissue distributions. Thus, the decrease in a portion of HU labeled 'atelectasis' does not necessarily mean opening of alveoli. Instead, edema fluid could be squeezed out of the lung and pushed into poorly aerated lung; however, we did not find changes in extravascular lung water [22] or lung tissue after the recruitment maneuver. Therefore, the observed changes in differently aerated lung volumes could have been caused by transformation of completely collapsed lung into partly opened lung or by an increased homogeneity in the distribution of alveolar fluid [25]. Importantly, the observed changes in aerated lung volume were relatively small 10 minutes after the recruitment maneuver and do not support the usefulness of such a maneuver, which has also been demonstrated in clinical studies [26]. Possibly higher levels of PEEP could have enhanced recruitment, but to avoid possible influences of PEEP on the physiological parameters studied we maintained the same level of PEEP (10 cmH_2_O).

### Evaluation of gas exchange parameters

Although impaired oxygenation is the main symptom in acute lung injury [27] correlated with atelectasis [28,29], our study suggests that PaO_2 _is less related to the amount of atelectatic lung than to the aerated lung that remains for ventilation. These studies suggested that there was a linear correlation between PaO_2 _or shunt and atelectasis formation, especially if atelectasis was below 5% of total lung [28]. Lung healthy subjects were studied, however, and only one slice of the lung close to the diaphragm was analyzed, representing the area where most atelectases occur. So atelectasis as a fraction of the whole lung was probably much lower. Furthermore, there seems to be a difference in the characteristic of atelectasis formation between otherwise healthy lungs and injured lungs with high proportions of instable alveolar units that are poorly ventilated. Poorly aerated lung has been considered as low  regions. Because we found a correlation between the PaO_2 _and poorly aerated lung, it is possible that the regional blood flow through these regions was considerably high. Therefore, intrapulmonary shunt does not only happen in totally collapsed, but also in low , units. What the clinician wants to know is whether a certain improvement in oxygenation can predict the amount of recruitment. Improvements in gas exchange after recruitment are attributed mainly to two basic mechanisms: first, by redirection of blood flow from nonaerated to aerated lung regions and reduction of venous admixture, which we observed; and second, which we did not observe, through an increase in alveolar ventilation, leading to a reduction in PaCO_2_. In several clinical studies that have failed to demonstrate a benefit for active recruitment [26,30,31], oxygenation parameters, but not mechanical parameters, were used for decision making. Because we could not find the PaO_2 _changes representative of recruitment, even in a very recruitable model, this could have important implications on the interpretation of these studies. It seems that the amount of oxygenation improvement is not so much determined by the reduction of nonaerated lung, but by the blood flow through these regions.

### Evaluation of respiratory mechanics parameters

The plateau pressure and static lung compliance correlated equally with nonaerated and poorly aerated lung volumes. It appears that in lung injury, V_POOR _and V_NON _are the main determinants in overall lung compliance. Following the argument of Barnas *et al*. [32] that the elastance (E) of the rib cage compartment is parallel to the elastance of the diaphragm-abdomen compartment, the elastances of the differently aerated lung compartments could behave similarly and thus be described by the equation 1/E_LUNG _= k_1_/E_HYP _+ k_2_/E_NORM _+ k_3_/E_POOR _+ k_4_/E_NON_, where the constants k_1–4 _depend on their fraction of total lung volume. Thus in healthy lungs, E_L _is mainly dependent on E_NORM_, because it has the highest fraction of lung volume. But with increasing fractions of E_POOR _and E_NON _(with much higher values than E_NORM_) they will become increasingly determinant for lung compliance. This hypothesis is supported by multiple regression analysis, showing that the fractional change of C_INF _was most dependent on V_POOR _(beta_S _0.550) and V_NON _(beta_S _-0.331).

The PV-curve has been used to obtain information about diseased lungs [33-36]. Although the calculated curve may not equally fit all data [37], the mathematical analysis of the PV-curve is objective and the best available algorithm so far [38]. Because the PV-curve characteristics reflect a dynamic investigation of the lung, they have been used to set the parameters of ventilation [39]. We did not investigate whether the point of maximum compliance increase really reflects the lower inflection point (LIP). We were surprised that the inflation point of maximum compliance increase actually increased after recruitment in a nonlinear way (Fig. 5d), with a sharp increase beyond an increase in aerated lung >20%. If the point of maximum compliance increase truly represented the commencement of alveolar recruitment, it should be lower in conditions with less atelectasis. An explanation for this phenomenon could be that recruitment happens throughout the inflation curve [36], making the existence of a singular threshold opening pressure unlikely. Also, inflation LIP has been shown to only poorly represent the pressure at which recruited lung stays open [33,40]. But since we did observe an increase in the LIP with recruitment, the logical consequence would be to increase PEEP after the recruitment maneuver.

Another parameter of the PV-curve, V_REC _has been used as an indicator of recruited volume in several investigations [36,41,42], but it had never been validated with actual CT measurements. Especially in ventilation with FiO_2 _1.0, the V_REC _represents unstable lung units prone to collapse. In our results, there was a significant increase in V_REC _after the recruitment maneuver, which correlated with the observed changes in V_POOR _and V_NON_. This means that a significant portion of the recruited lung still collapsed endexpiratory, probably because we did not increase PEEP after the recruitment. Therefore, V_REC _could not only serve as a measurement for recruited lung, but also for the lung in danger of being de-recruited.

## Conclusion

The findings of this study suggest that an improvement in oxygenation does not necessarily mean recruitment of nonaerated lung and that measures to recruit collapsed lung will have unpredictable results on gas exchange. The effects were diverse in magnitude and predicted changes in oxygenation and shunt did not correlate with alveolar recruitment. Poorly aerated lung regions were the main determinant for the observed changes in plateau pressure, respiratory system compliance and recruitable volume.

Lung recruitment might be grossly overestimated when simply looking at the PaO_2_. Also, the effects of a standard open-lung maneuver or currently advocated PEEP strategies on recruitment are relatively small [43]. Because we did not focus on optimal recruitment but on the relationship of certain parameters with changes in lung aeration, however, we used a recruitment procedure as proposed previously. Obviously, this specific recruitment maneuver was not sufficient to homogenize lung ventilation. Common treatment strategies in ARDS aim to improve oxygenation, and the mechanical properties of ventilator settings are adjusted according to gas exchange parameters (e.g. PEEP/FiO_2 _tables). The poor correlation we have found between oxygenation and recruitment might be a reason that several of these approaches have failed to show a benefit for the patients treated this way. We speculate that parameters other than gas exchange should be investigated as targets in treating these patients.

## Key messages

• 	The respiratory mechanics parameters correlated with the amount of aerated lung better than gas exchange parameters, with the venous admixture being the only oxygenation parameter that correlated with nonaerated lung volume.

• 	A recruitment maneuver without PEEP adjustment led to a decrease of nonaerated lung, presumably towards poorly aerated lung mainly. This did not significantly alter the distribution of a tidal breath to the differently aerated lung regions, however, implying that there was no reduction in the opening and collapse of alveoli.

• 	Changes in aerated and nonaerated lung volumes after the recruitment maneuver were adequately represented by changes in plateau pressure, respiratory system compliance and recruitable volume.

• 	An improvement in oxygenation does not necessarily mean recruitment of nonaerated lung and measures to recruit collapsed lung will have unpredictable results on gas exchange.

• 	In the clinical context, or even worse in clinical studies, using PaO_2 _changes as a surrogate for lung recruitment should be done with caution, as it lacks a clear physiological basis.

## Abbreviations

ARDS = acute respiratory distress syndrome; C_INF _= maximum inflation compliance; C_RS _= compliance of the respiratory system; CT = computer tomography; E = elastance; FiO_2 _= fraction of inspired oxygen; HU = Hounsfield unit; LIP = lower inflection point; PaO_2 _= arterial partial oxygen pressure; PEEP = positive end-expiratory pressure; PV-curve = (respiratory system) pressure volume curve; Q_VA_/Q_T _= venous admixture (according to Berggren's formula); RM = recruitment maneuver 45 cmH_2_O/40 s;  = ventilation-perfusion distribution; V_D_/V_T _= physiological dead space (according to Bohr/Enghoff's formula); V_GAS _= intrathoracic gas volume; V_HYP _= volume of hyperinflated lung parenchyma; V_NON _= volume of nonaerated lung parenchyma; V_NORM _= volume of normally aerated lung parenchyma; V_POOR _= volume of poorly aerated lung parenchyma; V_REC _= recruitable volume at end-expiration; V_TISS _= intrathoracic tissue volume.

## Competing interests

DH has received an unrestricted research grant in 2003 from Hamilton Medical Deutschland GmbH, by which the study was partially funded. All other authors declare that they have no competing interests.

## Authors' contributions

DH conceived the study, participated in the design and execution of the study, the analysis of data and finalized the manuscript. PP participated in analysis and interpretation of the data and revised the manuscript. RD participated in the animal experiments and the analysis of data. AU participated in the animal experiments and the analysis of multi-slice CT data. AM did the radiology studies and participated in the analysis of multi-slice CT data. RR participated in the study design and coordination and helped to draft the manuscript. RK participated in the study design, interpretation of results and writing of the manuscript.

## Supplementary Material

Additional File 1 Additional information on materials and methods. Click here for file

## Figures and Tables

**Table 1:** Lung volumes measured by multi-slice computer tomography

	Pre-recruitment maneuver	Post-recruitment maneuver	*P*-value	*fractional change *(%)
Expiration				
V_HYP _(ml)	60 ± 21	67 ± 28	0.025	11.2 ± 10
V_NORM _(ml)	577 ± 142	649 ± 206	0.036	11.0 ± 12
V_POOR _(ml)	406 ± 83	493 ± 112	0.017	21.7 ± 18
V_NON _(ml)	357 ± 53	275 ± 72	0.012	-23.3 ± 15
V_TOT _(ml)	1401 ± 136	1483 ± 175	0.025	5.8 ± 5
V_GAS _(ml)	629 ± 83	711 ± 133	0.012	13.1 ± 10
V_TISS _(ml)	838 ± 62	832 ± 60	0.263	-
Inspiration				
V_HYP _(ml)	109 ± 38	115 ± 42	0.093	-
V_NORM _(ml)	789 ± 140	889 ± 197	0.012	12.4 ± 12
V_POOR _(ml)	397 ± 94	478 ± 124	0.017	20.9 ± 18
V_NON _(ml)	295 ± 54	232 ± 75	0.012	-22.3 ± 16
V_TOT _(ml)	1589 ± 139	1713 ± 150	0.012	7.9 ± 5
V_GAS _(ml)	838 ± 84	939 ± 128	0.012	12.5 ± 8
V_TISS _(ml)	819 ± 56	838 ± 64	0.263	-

Data are reported as mean ± SD. V_GAS_, total lung gas volume; V_HYP_, hyperinflated lung volume; V_NON_, non-aereated lung volume; V_NORM_, normally aereated lung volume; V_POOR_, poorly aerated lung volume; V_TISS_, total lung tissue volume; V_TOT_, total lung volume.

**Table 2:** Gas exchange and hemodynamics parameters

	Pre-recruitment maneuver	Post-recruitment maneuver	*P*-value	*fractional change *(%)
PaO_2 _(mmHg)	71 ± 21	94 ± 28	0.017	33.0 ± 23
PaCO_2 _(mmHg)	81 ± 20	81 ± 19	0.575	-
PvO_2 _(mmHg)	45 ± 10	49 ± 10	0.093	-
Q_VA_/Q_T _(%)	50.2 ± 9.9	39.3 ± 8.6	0.036	-20.8 ± 16
V_D_/V_T _(%)	84 ± 2.9	83.7 ± 3.4	0.31	-
HR (min^-1^)	85 ± 84	77 ± 21	0.025	-11.3 ± 9
MAP (mmHg)	80 ± 15	83 ± 24	0.498	-
Q_T _(l min^-1^)	3.7 ± 0.2	3.4 ± 0.2	0.018	-9.6 ± 6
VO_2 _(ml min^-1^)	138 ± 39	141 ± 35	0.889	-
DO_2 _(ml min^-1^)	401 ± 118	412 ± 101	0.575	-
EVLWI (ml kg^-1^)	20.6 ± 7.9	21.1 ± 9.6	0.499	-

Data are reported as mean ± SD. DO_2_, oxygen delivery; EVLWI, extravascular lung water index; HR, heart rate; MAP, mean arterial pressure; PaCO_2_, arterial carbon dioxide partial pressure; PaO_2_, arterial partial oxygen pressure; , mixed venous partial oxygen pressure; Q_T_, cardiac output; Q_VA_/Q_T_, venous admixture; V_D_/V_T_, dead space fraction; VO_2_, oxygen consumption.

**Table 3:** Respiratory mechanics parameters

	Pre-recruitment maneuver	Post-recruitment maneuver	*P*-value	*fractional change*(%)
PIP (cmH_2_O)	36.6 ± 4	31.1 ± 3.7	0.012	-12.5 ± 6
P_PLAT _(cmH_2_O)	30.7 ± 3.1	27.2 ± 2.8	0.028	-13.8 ± 7
C_RS _(ml cmH_2_O^-1^)	13.5 ± 2.2	17.9 ± 2.6	0.028	34.5 ± 17
Pmci,_INF _(cmH_2_O)	22.4 ± 11.9	32.3 ± 5.4	0.046	113 ± 192
Pmcd,_INF _(cmH_2_O)	43.3 ± 9.5	56.6 ± 15.5	0.075	-
C_INF _(ml cmH_2_O^-1^)	24.4 ± 14.7	42.0 ± 14.5	0.028	101.8 ± 92
Pmci,_DEF _(cmH_2_O)	9.4 ± 2.2	9.9 ± 1.1	0.463	-
Pmcd,_DEF _(cmH_2_O)	19.9 ± 2.0	21.4 ± 1.9	0.046	7.0 ± 0.7
V_REC _(ml)	183 ± 135	256 ± 145	0.028	66.5 ± 47

Data are reported as mean ± SD. C_INF_, maximum inflation compliance; PIP, peak inspiratory pressure; P_PLAT_, plateau pressure; C_RS_, static respiratory system compliance; Pmci,_DEF_, point of maximum compliance increase of deflation curve; Pmcd,_DEF_, point of maximum compliance decrease of deflation curve; Pmcd,_INF_, point of maximum compliance decrease of inflation curve; Pmci,_INF_, point of maximum compliance increase of inflation curve; V_REC_, recruitable volume at 10 cmH_2_O.
