# Supplementary material for: Respiratory compliance but not gas exchange correlates with changes in lung aeration after a recruitment maneuver: an experimental study in pigs with saline lavage lung injury
Source: Crit Care. 2005 Jul 13;9(5):R471–82. doi: 10.1186/cc3772 (PMC1297611; doi:10.1186/cc3772)
Supplement: Additional File 1 — Additional information on materials and methods. [file cc3772-S1.doc]

# Online data supplement

## **METHODS AND MATERIALS**

## *Animal preparation.* Anesthesia was induced intravenously with pentobarbital and ketamine in premedicated female pigs (race: DL), followed by a continuous infusion of propofol, fentanyl and pancuronium (see Table 1). Animals were orotracheally intubated and ventilated in constant flow, volume controlled mode with FiO_2_ 1.0 throughout the protocol. Tidal volume was set to 8 ml/kg body weight, the initial respiratory rate to achieve a PaCO_2_ of 35-45 mmHg with an I:E ratio of 1:1 and the PEEP was set to 10 cmH_2_O throughout the experiment. A 4 Fr. catheter (Pulsion, Munich, Germany) was inserted into the femoral artery and a 8.5 Fr venous sheath (Arrow, Erding, Germany) into the femoral vein. A pulmonary artery catheter (Becten Dickinson, Heidelberg, Germany) was positioned under transduced pressure guidance. Since animals were fasted a bolus of 500 ml hydroxyethyl starch 200/0.5 was given and a continuous infusion of Ringer’s solution started at 5 ml/kg h^-1^. Urine output was measured by transurethral catheterisation.

For induction of lung injury, aliquots of 1500 ml of warmed saline (38°C) were instilled via the tracheal tube and kept for 30 seconds or if SpO_2_<80% or the mean pulmonary artery pressure (MPAP) increased >50%, whichever came first. Between lavages 2-5 minutes were allowed for stabilization, until SpO_2_>90%. ALI was considered to be stable when there was a constant PaO_2_ below 100 mmHg for at least 60 minutes.

### *Cardiovascular, ventilatory and oxygenation measurements.* Blood pressures were directly measured, with the zero reference point set at midchest level. Cardiac output (Q_T_) was determined by pulmonary arterial thermodilution technique as the mean of three measurements taken at end-expiration and calculated by a cardiac output computer (S5 compact monitor, Datex-Ohmeda, Duisburg, Germany). If measurements differed >10% between each others, two more measurements were obtained and both extremes discarded. Systemic (SVR) and pulmonary vascular resistance (PVR) were calculated using standard formulas. An inline temperature sensor was attached to the central venous catheter and connected together with the thermistor of the arterial catheter to a PiCCO computer (Pulsion, Munich, Germany). By transcardiopulmonary thermodilution intrathoracic blood volume (ITBV) and extravascular lung water (EVLW) were determined and indexed to body weight.

Esophageal pressure (P_ES_) was measured by a balloon catheter (International Medical, Allegiance, Kleve, Germany) connected to the AUX port of the ventilator and correct placement was verified using the occlusion technique (1). Before each recording the balloon was emptied and refilled with 1 ml of air. Transpulmonary pressure P_TP_ was calculated as airway opening pressure (P_AW_) minus P_ES_. Ventilator settings and measurements were recorded with a sampling rate of 70 Hz on a computer connected to the RS232 port of the ventilator (Datalogger 3.27, Hamilton).

### *Expiratory volume correction.* Gas flow and airway pressure (P_AW_) were measured between the tracheal tube and the Y-piece of the ventilator circuit by a membrane differential pressure transducer, connected to the ventilator (Galileo Gold, Hamilton Medical, Rhäzüns, Switzerland, accuracy ±5 %). V_T_ was derived from the integrated flow signal. Expiratory volumes were corrected as proposed by Jonson et al (2). If expiratory volumes are not corrected for different gas composition, intrapulmonary humidification and temperature, expiratory tidal volumes differ from inspired volumes at steady state ventilation. The flow calibration factor for all expiratory volumes was calculated, that at steady state, expired volumes became identical to inspired volumes. For exemplary validation, expiratory tidal volume (VT_EX_) was corrected on the basis of Boyle’s law to inspiratory conditions as proposed by Gattinoni et al (3):

$$VT_{CORR}=VT_{EX}\left(\frac{273+T_{A}}{273+T_{B}}\right)\times\left(\frac{P_{B}-P_{H2O}}{P_{B}}\right)$$

for T_A_ = ambient temperature, T_B_ = body temperature, P_B_ = barometric pressure, P_H2O_ = vapor pressure at ambient temperature. Correlation with inspiratory tidal volume (VT_IN_) was excellent (r²=0.921, p<0.0001), the mean values for all measurements were VT_EX_ = 249±17 ml, VT_CORR_ = 232±15 and VT_IN_ = 230±16 ml. There was no difference between VT_IN_ and VT_CORR_ (p=0.346), but between VT_IN_ and VT_EX_ (p<0.0001).

Expired gas was collected in a 10 L box heated to 37° to maintain BTPS conditions. Mixed expired gaseous samples were taken from this, corrected for ventilator flow-by and used for calculation of physiologic dead space (V_D_/V_T_) according to the modified Bohr equation:

*Oxygenation parameters.* Arterial and mixed venous blood samples were collected simultaneously and analyzed immediately for blood gases and hemoglobin (Abl 510, Radiometer, Copenhagen, Denmark). Oxygen saturated hemoglobin (HbaO_2_ and ) was measured with a species adjusted co-oximeter (OSM3, Radiometer). Oxygen concentrations were calculated from these and venous admixture (Q_VA_/Q_T_) determined using the shunt equation (4).

#### Lung mechanics measurements. Compliances were computed as follows(5)(see Figure 1):

static compliance of respiratory system C_RS_ = V_T_ /(Paw_2_-Paw_ex_)

static compliance of chest wall C_W_ = V_T_/(Pes_2_-Pes_ex_)

static compliance of lung C_L_ = V_T_/[(Paw_2_-Paw_ex_)-(Pes_2_-Pes_ex_)]

#### Analysis of PV-curves. An inflation-deflation quasi-static pulmonary pressure-volume (PV-) curve starting from ZEEP was performed by a new algorithm implemented into the ventilator (PV-Tool, Galileo Gold, Hamilton Medical, Rhäzüns, Switzerland). Every PV-maneuver was preceded by a prolonged exhalation to ZEEP to standardize lung volume history. Lungs were inflated with a constant pressure rise of 2 cmH_2_O/sec up to 45 cmH_2_O, keeping a plateau at 35 cmH_2_O for 1 sec and deflating with the same rate with a 0.6 sec pause every 5 cmH_2_O downwards to preset PEEP. Total maneuver time amounted to 54 sec. Expiratory volumes were corrected as described. The correction factor was calculated from the Vt_in_/Vt_ex_ ratio during normal tidal ventilation before each maneuver. Inflation and deflation curves were separated and analyzed each by using the Levenberg-Marquard iterative algorithm fitted to the following equation (6):

$dV=a+\frac{b}{1+e^{-(P-c)/d}}$

with the initial fitting parameters a=0, b=500, c=20, d=10 and the residual sum of squares was calculated. The fitting regression was stopped automatically after a maximum of 100 iterations and judged as non-conversion for this case. The pulmonary PV-curve did not converge to the fitting algorithm in all the curves of 2 animals, leaving 6 experiments for analysis of PV-curves. The goodness of fit in these curves was good as depicted by R², which was 0.998±0.003 for inflation and 0.986±0.023 for deflation curves. A graphically assessable LIP was present only in 80% and a UIP in 31% of all curves, so the mathematically derived inflection points were used for further analysis. The equation represents a sigmoid shape, which has been shown to fit experimental and clinical pressure-volume curves with great accuracy (7). The fitting parameters have a physiological meaning, with a = lower asymptote and lung relaxing volume to start with, b = upper asymptote representing TLC, c = pressure at the steepest part of the curve (= maximum compliance, C_MAX_) and d = measure for the spread of the curve, from which inflection points can be easily calculated as

pressure at maximum compliance increase P_mci_ = c – d × 1.317 ≈ lower inflection point

pressure at maximum compliance decrease P_mcd_ = c + d × 1.317 ≈ upper inflection point

From a mathematical point of view these are the maximum values of the 2^nd^ derivate and represent clearly identified points where a rapid change in slope takes place, clinically often referred to as lower and upper flexion points.

REFERENCES

1. Baydur A, Behrakis PK, Zin WA et al. A simple method for assessing the validity of the esophageal balloon technique. *Am Rev Respir Dis* 1982; 126:788-791

2. Jonson B, Beydon L, Brauer K et al. Mechanics of respiratory system in healthy anesthetized humans with emphasis on viscoelastic properties. *J Appl Physiol* 1993; 75:132-140

3. Gattinoni L, Mascheroni D, Basilico E et al. Volume/pressure curve of total respiratory system in paralysed patients: artefacts and correction factors. *Intensive Care Med* 1987; 13:19-25

4. Berggren SM. The oxygen deficit of arterial blood caused by non-ventilated parts of the lung. *Acta Physiol Scand Suppl* 1942; 4:4-92

5. D'Angelo E, Robatto FM, Calderini E et al. Pulmonary and chest wall mechanics in anesthetized paralyzed humans. *J Appl Physiol* 1991; 70:2602-2610

6. Venegas JG, Harris RS, Simon BA. A comprehensive equation for the pulmonary pressure-volume curve. *J Appl Physiol* 1998; 84:389-395

7. Harris RS, Hess DR, Venegas JG. An objective analysis of the pressure-volume curve in the acute respiratory distress syndrome. *Am J Respir Crit Care Med* 2000; 161:432-439

**Figure legends**

**Figure 1**: Original trace for airway and esophageal pressure during lung mechanics measurement

**Figure 2**. Original data of inflation PV values and fitting curve.

(continuous line: calculated curve; dots: measured data)

**Figure 3**. Experimental protocol, original trace from animal 8 a) airway and esophageal pressures in cmH_2_O b) flow in ml/s c) volume in ml. Abscisse in seconds. Left side pre recruitment maneuvre (RM) of 3 times sustained inflation with 45 cmH_2_O for 40 sec. (not displayed), right side post RM; Note increase in inspiratory (V_MAX_) and expiratory volume (V_REC_) during PV-maneuvre after the RM in c).

###### Figure 4 Inflation and deflation PV-curve of an exemplary animal before and after the recruitment maneuver.

## **Table 1**: Fluid balance and sedation regimen

|  | pre RM  mean±SD | post RM  mean±SD |
| --- | --- | --- |
| fluid intake [ml kg^-1^ h^-1^]  (since start of experiment) | 9.3±1.7 | 9.3±1.6 |
| fluid balance [ml kg^-1^ h^-1^] (since start of experiment) | +2.4±4.0 | +2.6±3.4 |
| Propofol [mg kg^-1^ h^-1^] | 7.7±1.7 | 7.7±1.7 |
| Fentanyl [mcg kg^-1^ h^-1^] | 8.0±2.2 | 8.0±2.2 |

## RM = recruitment maneuver

## **Table 2**. Additional hemodynamic data

|  | pre recruitment maneuver | post recruitment maneuver | *P*-value |
| --- | --- | --- | --- |
| CVP [mmHg] | 7±3 | 8±3 | 0.084 |
| MPAP [mmHg] | 34±9 | 33±10 | 0.463 |
| PCWP [mmHg] | 9±3 | 9±3 | 0.458 |
| SVR [dyn sec^-1^ cm^-5^] | 1597±368 | 1765±438 | 0.05 |
| PVR [dyn sec^-1^ cm^-5^] | 563±214 | 573±240 | 1.0 |
| SV [ml] | 45±10 | 45±9 | 0.889 |
| ITBVI [ml kg^-1^] | 16.2±5 | 16.9±5.2 | 0.398 |

CVP = central venous pressure; PCWP = pulmonary capillary wedge pressure; MPAP = mean pulmonary artery pressure; SVR and PVR = systemic and pulmonary vessel resistance; SV = stroke volume; ITBVI = intrathoracic blood volume index.

**Figure 1.**

**Figure 2**

### Figure 3


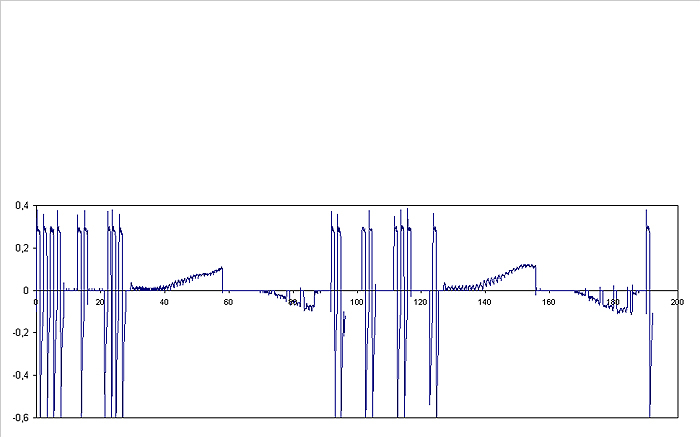

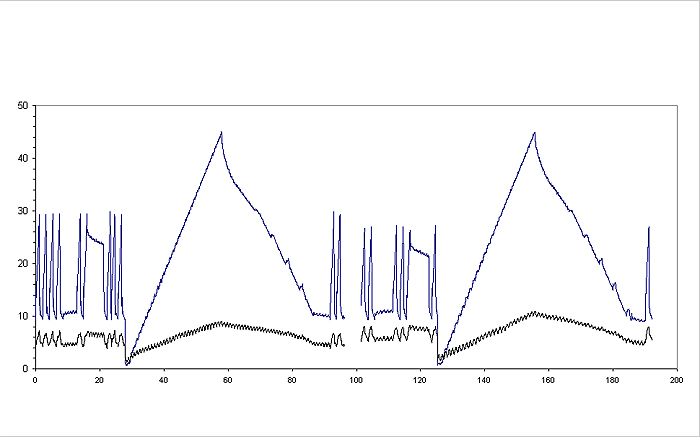

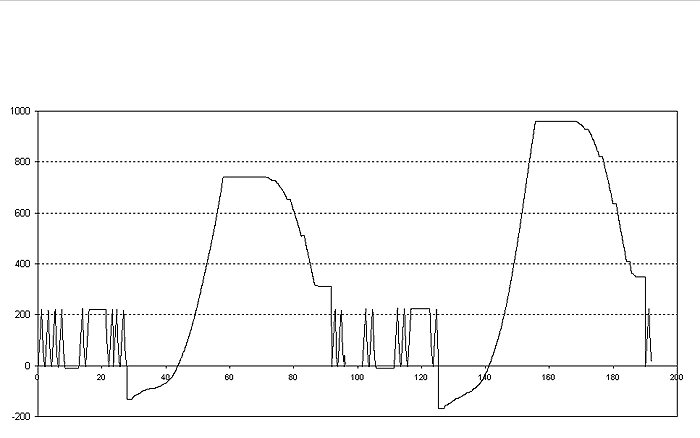


**3 x recruitment maneuvers (RM) 45 cmH_2_O for 40 seconds**

**V_MAX_**

**V_MAX_**

**V_REC_**

**V_REC_**

**P_MAX_**

**a) PRESSURE**

**b) FLOW**

**c) VOLUME**

**Figure 4**
